# Supplementary material for: Intravital imaging of the formation and resolution of MHC class II–positive T-cell activation niches
Source: Life Sci Alliance. 2026 Jan 2;9(3):e202503476. doi: 10.26508/lsa.202503476 (PMC12759086; doi:10.26508/lsa.202503476)
Supplement: Supplementary file 1 [file LSA-2025-03476_TableS1.docx]

**Supplemental Table S1. Two-photon microscope parameters**

| **Channel** | **Laser** | **Dichroic mirror** | **Bandpass filter** | **1° Fluor** |
| --- | --- | --- | --- | --- |
| **1** | Mai Tai / 800nm | 509nm | 519-549 | mAmetrine |
| **2** | Insight / 950nm | 509nm | 420-500 | SHG |
| **3** |  | 650nm | 575-630 | LSSmOrange |

| **Channel** | **Laser/excitation** | **Dichroic mirror** | **Bandpass filter** | **1° Fluor** | **2° Fluor*** |
| --- | --- | --- | --- | --- | --- |
| **1** | Mai Tai / 790nm | 509nm | 420-500 | TagBFP |  |
| **2** |  | 509nm | 519-549 | mAmetrine | TagBFP |
| **3** |  | 640nm | 573-613 | TagRFP | mAmetrine |
| **4** |  | 640nm | 634-686 | TagRFP or AF647 |  |
| **5** | Insight / 1010nm | 509nm | 420-500 | SHG |  |
| **6** |  | 509nm | 519-549 | Venus |  |
| **7** |  | 640nm | 573-613 | OFP | Venus, TagRFP |
| **8** |  | 640nm | 634-686 |  | OFP |
|  |  |  |  |  |  |

*2° Fluor, bleed over emission in this channel that was removed by spectral unmixing.
