# Supplementary material for: Intravital imaging of the formation and resolution of MHC class II–positive T-cell activation niches
Source: Life Sci Alliance. 2026 Jan 2;9(3):e202503476. doi: 10.26508/lsa.202503476 (PMC12759086; doi:10.26508/lsa.202503476)
Supplement: Supplementary file 2 [file LSA-2025-03476_TableS2.docx]

**Supplemental Table S2. IEbeta-mAmetrine integration site**

AGTTGCAACTGGATCCAGCATTTTAAAAAAGAAAATAAATTGATCCTTTAAAAAATAATATATAATTTATACCCAGAAGAAGTTATATGTGTATACAATGTATTTTGAGCCTATCCACCACCCACTACCTCCTTCCAGCTTCTTTTGGAACCTTCCAATAATGTCTCCCTCCCAAATTCATTTCCTCTTTAAACAACAACAACAACAACAACAACAACAACAAAAAACCAAAAAAACACACACACACAAACAAACAAACAAACAACCCAAAGCAAAACCCTACTCTCCTTTTGTTTGTTTGGTTTTTGTTGTTGTTTATTTGTTTGTTAATTAGAGACAGAATCTTACTATGTAGAGCAGGCTGACCTTTCTATCTCACAGAGATCCAGCTGCCTCTGCCTCCTGAGTGCTGGGATATGAGGCATGGGCCAGCAGCCCAGACTGAGTATCCATGTAATGAAGAGAACTGCAAGTTTCAGAAGGGGACCTGCAAACTGAATCTCTAACTAGCAACTGATGATGCTGGACTCCTTTGATGCTGATTGGCTCCCAGCACTGGCCTTACCCAATCCAGTGGCAAAGCAGTGAATGCCCTGTCTCTTATTATCTTAGCAATGAGTAAAGAGAATAAAGTTACAGTCTGAAGCTTGCCTTCCCCTCTGACTCCTGTGTCTCCTCTCCTGCAGC**gccgccacc**ATGGTGAGCAAGGGCGAGGAGCTGTTCACCGGGGTGGTGCCCATCCTGGTCGAGCTGGACGGCGACGTAAACGGCCACAAGTTCAGCGTGCGCGGCGAGGGCGAGGGCGATGCCACCAACGGCAAGCTGACCCTGAAGTTCATCTGCACCTCCGGCAAGCTGCCCGTGCCCTGGCCCACCCTCGTGACCACCCTGTCTTACGGCGTGCAGTGCTTCGCCCGCTACCCCGACCACATGAAGCAGCACGACTTCTTCAAGTCCGCCATGCCCGAAGGCTACGTCCAGGAGCGCACCATCTCCTTCAAGGACGACGGCAGCTACAGGACCCGCGCCGAGGTGAAGTTCGAGGGCGACACCCTGGTGAACCGCATCGAGCTGAAGGGCATCGACTTCAAGGAGGACGGCAACATCCTGGGGCACGCTGGAGTACAACATGAACGTGTGGGACGCGTATATCACGGCCGACAAGCAGAAGAACGGCATCAAAGCGAACTTCAAGATCGAGCACAACGTCGAGGACGGCGGCGTGCAGCTCGCCGACGCGTACCAGCAGAACACCCCCATCGGCGACGGCTCCGTGCTGCTGCCTGACAACCACTACCTGAGCTTCCAGAGCAAGCTGTTCAAAGACCCCAACGAGCAGCGCGATCACATGGTCCTGCTGGAGTTCGTTACCGCCGCCGGGATCACTCCCGGCATGGACGAGCTGTACAAGTAATCCTCAGGTGCAGGCTGCCTATCAGAAGGTGGTGGCTGGTGTGGCCAATGCCCTGGCTCACAAATACCACTGAGATCTTTTTCCCTCTGCCAAAAATTATGGGGACATCATGAAGCCCCTTGAGCATCTGACTTCTGGCTAATAAAGGAAATTTATTTTCATTGCAATAGTGTGTTGGAATTTTTTGTGTCTCTCACTCGGAAGGACATATGGGAGGGCAAATCATTTAAAACATCAGAATGAGTATTTGGTTTAGAGTTTGGCAACATATGCCCATATGCTGGCTGCCATGAACAAAGGTTGGCTATAAAGAGGTCATCAGTATATGAAACAGCCCCCTGCTGTCCATTCCTTATTCCATAGAAAAGCCTTGACTTGAGGTTAGATTTTTTTTATATTTTGTTTTGTGTTATTTTTTTCTTTAACATCCCTAAAATTTTCCTTACATGTTTTACTAGCCAGATTTTTCCTCCTCTCCTGACTACTCCCAGTCATAGCTGTCCCTCTTCTCTTATGGAGATCC**CCAGAGTTCCCTGTGTGGCAGCTGTGATCCTGTTGCTGACAGTGCTGAGCCCTCCAGTGGCTTTGGTCAGAGACTCCAGAC**GTAAGTGCACACCTCAGGTGCTGGGATGCTCGGGGTCGGGGAAGGAAGGAGCTAACATTCTCACTGTCCAGGCCAAGTCCCTCGGAACTATTGATATCTTCTGTGAGCATGCACAGTCCTCACATGAACTCTAAACTATGTCCCCAAACAGAAGCCTGGATGTTTGTGCTCTCAGATCTGTGTGAGAGGCCGCTGGGTATCAGCCATTGCTTTTCAAGTTTCTCCCAGTAACATCTACACCTGCGTCATATTCCTTTAGGGCCCTGAGAAGATCGATGAATCATGATGTTAAATTAACTTCTGGGACAGGCCCTGTCTTCACTGTGTTGAGATAAAGCCAGACAAAGTGGTTTCTCTTTAAAAATAGAACTTCCTATGTGTGTTTCTATTCTACTTTTTATGTATGTTTTCTGTTGTTT

The sequence of the IEbeta-mAmetrine integration site is show. The 5’UTR (red), the sgRNA homology sequence (yellow highlight), the coding region of Exon 1 (blue), and the first intron (red) of the endogenous IEbeta gene are shown. The Kozak sequence (lower case, bold), the mAmetrine coding region (underline) and the rabbit beta globin 3’UTR/poly A addition site are shown in green.
