# Supplementary material for: Intravital imaging of the formation and resolution of MHC class II–positive T-cell activation niches
Source: Life Sci Alliance. 2026 Jan 2;9(3):e202503476. doi: 10.26508/lsa.202503476 (PMC12759086; doi:10.26508/lsa.202503476)
Supplement: Supplementary file 3 [file LSA-2025-03476_TableS3.docx]

**Supplemental Table S3. CD8-LSSmOrange integration site**

TCAAGAAGCAATGTCACTCAAAACTAGGAGGTTTTAGCATTTTTTTTTCTTTTTTAAACATTTCTTTATTTTGTGTATACACATGCATTGTGGTAGGGCAGAATAACTAGTACTGGGTATTGAGCTCAGATCATACTTGGAAGTAAAAGCACCTCTACTCACTGAGACATCCTACTGGTCCCAAAGATAGTAGTTTTGAGCCCTAGTGTGTGTGTGTGTGTGTGTGTGTGTGTGTGTGTGTGTGTGTGTATACATCTGTGTGTCTCTATATGTTATTTTGTTGTAGTTTGGTTTTCTTTGTTGTTGGTTTGGTTTGGTTTTTTTAAACTTTAAGGTTTAAAATGCTAGAAGTGGGCCTTACAAAAACCATCCTAACAGAGCTCTCAGCCCCAGGCATGCCCTCCTCTTCCTCCATCTGTCATTGCTTTTGCTATAAGAAATATAGCAAAAGTGTCACTGGCCTGAAAATAAGAGTCTTACAATTTGCTGTTGTTTCCCAG**GTTTCACAAATGA**TCAGCCCCCCTCTCCCTCCCCCCCCCCTAACGTTACTGGCCGAAGCCGCTTGGAATAAGGCCGGTGTGCGTTTGTCTATATGTTATTTTCCACCATATTGCCGTCTTTTGGCAATGTGAGGGCCCGGAAACCTGGCCCTGTCTTCTTGACGAGCATTCCTAGGGGTCTTTCCCCTCTCGCCAAAGGAATGCAAGGTCTGTTGAATGTCGTGAAGGAAGCAGTTCCTCTGGAAGCTTCTTGAAGACAAACAACGTCTGTAGCGACCCTTTGCAGGCAGCGGAACCCCCCACCTGGCGACAGGTGCCTCTGCGGCCAAAAGCCACGTGTATAAGATACACCTGCAAAGGCGGCACAACCCCAGTGCCACGTTGTGAGTTGGATAGTTGTGGAAAGAGTCAAATGGCTCTCCTCAAGCGTATTCAACAAGGGGCTGAAGGATGCCCAGAAGGTACCCCATTGTATGGGATCTGATCTGGGGCCTCGGTGCACATGCTTTACATGTGTTTAGTCGAGGTTAAAAAACGTCTAGGCCCCCCGAACCACGGGGACGTGGTTTTCCTTTGAAAAACACGATGATAATATG**gccacagcc**ATGGTGAGCAAGGGCGAGGAGAATAACATGGCCATCATCAAGGAGTTCATGCGCTTCAAGGTGCGCATGGAGGGCTCCGTGAACGGCCACGAGTTCGAGATCGAGGGCGAGGGCGAGGGCCGCCCCTACGAGGGCTTTCAGACCGTTAAGCTGAAGGTGACCAAGGGTGGCCCCCTGCCCTTCGCCTGGGACATCTTGTCCCCTCAGTTCACCTACGGCTCCAAGGCCTACGTGAAGCACCCCGCCGACATCCCCGACTACCTCAAGCTGTCCTTCCCCGAGGGCTTCAAGTGGGAGCGCGTGATGAACTTCGAGGACGGCGGCGTGGTGACCGTGACTCAGGACTCCTCCCTGCAGGACGGCGAGTTCATCTACAAGGTGAAGCTGCGCGGCACCAACTTCCCCTCCGACGGCCCCGTAATGCAGAAGAAGACCATGGGCATGGAGGCCTCCTCCGAGCGGATGTACCCCGAGGACGGCGCCCTGAAGGGCGAGGACAAGCTCAGGCTGAAGCTGAAGGACGGCGGCCACTACACCTCCGAGGTCAAGACCACCTACAAGGCCAAGAAGCCCGTGCAGTTGCCCGGCGCCTACATCGTCGACATCAAGTTGGACATCACCTCCCACAACGAGGACTACACCATCGTGGAACAGTACGAACGCGCCGAGGGCCGCCACTCCACCGGCGGCATGGACGAGCTGTACAAGTAACACTGATAGCCTGCTAGCAATAAAAGGACTGTAACTCAATCAAAGCAAGATTTGAACTAGTGAGAGACAGCGCATTCAAGACTGGAGAGGCCCTTGCCCTTGCTCAAGCTGCCTGCTTTGAACTGCTGCAAGCTCTGTGTGTGGTCTGGGGTCTCACAGGGAAGGCCCCAGAACCCAAGCTCACTCACAGAGTGGCTGAAGAACCAAGAACTCCCACACCACCATGTACTCTTCTTGGACTGGGCCTTGGACAGTGGCCTTCCAGCCACTGTCTTTGTCAGTTGCTTGGTATGACAGCCTTATTGACTCTTTGGCCTTTCATGGAAAAGAGCTAGCCGGGACCCTGGAGAAGACTGCAAATCCTCACACAGAGAAGCCACCTCATGAATAAAGTTTTCTGCAAACAGTTTCAGGGCTCTCAGCAGCCATCTTACTCTCTCAGGCCTTGCCGTGGGTTTGGGAGGCTGTTGGCTCAGGAGACAGACTTCTACTTCATCTCCTAGTAGGAAGTTTCTAAGCTTCACTTCTTCTCTCTCGTGGCTTATATATTTCTTCTCTCACTCTCTGCCCTTTT

The sequence of the CD8beta-LSSmOrange integration site is shown. Intron 5 (red), the coding region of Exon 6 including the termination codon (blue), the sgRNA homology sequence (yellow highlight), and the 3’UTR (red) of the endogenous CD8beta gene are shown. The IRES, Kozak sequence (lower case, bold), and LSSmOrange coding region (underline) are shown in green.
